# Use of infrared thermography in assessing the thermal comfort of cattle–systematic review

**DOI:** 10.3389/fvets.2026.1788172

**Published:** 2026-05-18

**Authors:** Vanessa Sousa Pinto, Luís Gustavo Paixao Vilela, Italo Messias Ferreira de Souza, Carlos Eduardo Lima Sousa, Thiago Nogueira da Silva, Alinne da Silva Souza, Cláudio Vieira de Araújo, Marina de Nadai Bonin Gomes, Lucietta Guerreiro Martorano, Raimundo Nonato Colares Camargo-Júnior, Kedson Alessandri Lobo Neves, Lilian K1tia Ximenes Silva, Eder Bruno Rebelo da Silva, Welligton Conceiçao da Silva

**Affiliations:** 1 Postgraduate Program in Animal Science, Institute of Agricultural and Environmental Sciences, Federal University of Mato Grosso (UFMT), Sinop, Mato Grosso, Brazil; 2 Academics of the Veterinary Medicine Course at the University Center of the Amazon (UNAMA), Santarém, Pará, Brazil; 3 Postgraduate Program in Animal Science - Federal University of Mato Grosso do Sul. UFMS, Campo Grande, Mato Grosso do Sul, Brazil; 4 Embrapa Eastern Amazon, Santarém, Pará, Brazil; 5 Federal Institute of Education, Science and Technology of Pará (IFPA), Santarém, Pará, Brazil; 6 Institute of Engineering and Geosciences, Federal University of Western Pará (UFOPA), Santarém, Brazil; 7 Institute of Veterinary Medicine, Federal University of Pará (UFPA), Castanhal, Pará, Brazil; 8 Postgraduate Program in Animal Science (PPGCAN), Institute of Veterinary Medicine, Federal University of Pará (UFPA), Castanhal, Pará, Brazil

**Keywords:** environmental indices, heat stress, thermal comfort, thermal windows, thermoregulation

## Abstract

Heat stress is one of the main challenges of cattle farming in tropical and subtropical regions, affecting productivity, reproduction and welfare. With the increase in the frequency and intensity of heat waves, efficient monitoring becomes essential. Traditional physiological methods, such as rectal temperature and respiratory rate, although effective, are invasive and require restraint, limiting large-scale use. In this context, infrared thermography (IRT) emerges as a non-invasive and agile technique to measure surface temperature and identify changes related to thermoregulation. Therefore, the objective of this study was to describe the use of infrared thermography in assessing thermal comfort in cattle (*Bos taurus, Bos indicus*, and their crossbreeds). This literature review, conducted between 2020 and 2025 in the ScienceDirect and PubMed databases, was guided by PRISMA and composed of 83 articles, aimed to synthesize evidence on the applications, thermal windows, and limitations of IRT in cattle. The results indicate that orbital, nasal, vulvar and udder regions are more reliable for correlating surface temperature with classical environmental indices. IRT has been applied to lactating dairy cows, beef cattle under silvopastoral systems and integrated with artificial intelligence algorithms. Despite methodological and environmental limitations, IRT is consolidated as a promising tool for management, sustainability and welfare decisions in livestock.

## 1 Introduction

The progressive increase in global temperatures and the greater frequency of heat waves have intensified the challenges related to thermal management in cattle, particularly in tropical and subtropical regions. Under these conditions, the ability of animals to maintain homeothermy becomes compromised, directly affecting productivity, reproductive efficiency, immune status, and animal welfare (1, 2).

Heat stress can be defined as a condition in which the total heat load, resulting from metabolic heat production combined with environmental heat load, exceeds the animal's capacity for adequate heat dissipation. This imbalance triggers physiological disturbances and activates compensatory mechanisms aimed at maintaining thermal homeostasis (3, 4).

The thermoneutral zone in cattle varies according to species, production level, and environmental conditions. It is frequently described between approximately 5 °C and 25 °C for *Bos taurus*, whereas *Bos indicus* cattle exhibit greater tolerance to elevated temperatures and may maintain physiological stability at values close to 28–30 °C (1, 3, 5).

Environmental indices such as the Temperature–Humidity Index (THI) are widely used to estimate thermal risk in cattle, with values close to 68 often considered indicative of the onset of heat stress, and progressive aggravation occurring at values above 72–78, depending on animal category and production system (3, 4, 6). The Black Globe Humidity Index (BGHI) incorporates solar radiation through black globe temperature, allowing a more comprehensive evaluation of radiant heat load, particularly in pasture-based systems (7, 8).

Although THI and BGHI integrate relevant climatic variables, studies indicate that these indices do not replace the direct assessment of individual physiological responses, which may vary according to genetics, production category, and management conditions (3, 6). Conventional methods such as rectal temperature and respiratory rate provide good accuracy; however, they require animal restraint and handling, which may influence behavior and limit their applicability in large-scale production systems (9, 10).

In this context, infrared thermography (IRT) emerges as a non-invasive, sensitive, and rapidly applicable tool capable of measuring body surface temperature through the detection of infrared radiation emitted by the skin. The technique allows visualization of thermal variations in specific anatomical regions, reflecting vasomotor changes associated with peripheral blood flow redistribution during thermoregulatory processes (2, 7).

Beyond livestock production, IRT has been widely employed in biomedical and translational research, demonstrating high sensitivity in detecting microcirculatory alterations and physiological responses to stress, which reinforces its methodological robustness and scientific applicability (11).

In farm animals, recent reviews highlight the effectiveness of IRT in identifying thermal alterations associated with heat stress (2). The selection of appropriate “thermal windows” is fundamental for measurement accuracy. Regions such as the orbital, nasal, vulvar, and mammary areas are frequently described as more sensitive to microclimatic variations due to greater peripheral vascularization and lower interference from hair coverage (5, 6). Studies indicate that these areas show consistent correlations with environmental indices such as THI and BGHI, enabling integrated assessment between environmental conditions and physiological responses (12).

Studies conducted in tropical, silvopastoral, and feedlot systems indicate that infrared thermography enables the identification of thermal patterns associated with shade availability, ventilation, and solar radiation, contributing to management adjustments and greater productive resilience in the face of climate change (8, 13).

The intensification of global warming further reinforces the need for real-time thermal monitoring tools in livestock production. Recent studies addressing the impacts of climate change on ruminants highlight those non-invasive technologies, such as IRT, represent strategic instruments to support adaptive decision-making and promote more sustainable production systems (14).

Despite its considerable potential, IRT presents limitations related to coat characteristics, skin pigmentation, environmental conditions, and the lack of methodological standardization. Factors such as direct solar radiation, wind speed, relative humidity, capture distance, and camera angle directly influence measurement accuracy, requiring rigorously defined protocols and proper equipment calibration (10, 15).

In this context, a relevant scientific gap remains regarding protocol standardization and the identification of the most consistent thermal windows for indicating thermal discomfort, particularly under tropical conditions. Therefore, this study seeks to address three central questions: (1) how has infrared thermography been applied in the evaluation of thermal comfort in cattle (*Bos taurus, Bos indicus*, and crossbreeds)? (2) what are its main advantages and limitations? and (3) which methodological and scientific gaps still limit its standardized adoption? By clarifying these aspects, this review aims to strengthen the understanding of the practical applicability of IRT and to guide future research toward improving thermal monitoring strategies in livestock production systems.

## 2 Materials and methods

This systematic review followed the PRISMA 2020 guidelines (16), with the objective of identifying and analyzing studies that used IRT in the evaluation of thermal comfort in cattle published between 2020 and 2025. The review sought to answer three central questions: how IRT has been applied in the thermal monitoring of cattle, which thermal and variable windows have the greatest correlation with its results, and what methodological limitations are reported in these studies.

The time frame from 2020 to 2025 was chosen because this period concentrates greater technological evolution in thermal imaging cameras. There was an expansion in the use of portable equipment, improvement in the accuracy of images and greater integration of IRT with precision livestock tools. In addition, recent studies present more standardized methodologies, which allows for more consistent comparisons between surveys.

The search was carried out between January and March 2025 in the PubMed, ScienceDirect, Scopus, Web of Science, and Google Scholar databases. Keywords combined with Boolean operators were used: (“infrared thermography” OR “thermal imaging”) AND (cattle OR bovine OR “*Bos indicus*” OR “*Bos taurus*”) AND (“heat stress” OR “thermal comfort” OR “heat load”). Filters were applied to select articles published in the defined period, written in English and Portuguese and classified as original studies.

Studies that used IRT as the main tool for thermal measurement in cattle of the species *Bos taurus, Bos indicus* or crosses were included. Only studies that presented complete experimental data and related IRT with physiological, behavioral, or environmental variables were also considered. Studies with other species, articles without clear methodology, studies that used IRT only as an illustration, studies focused on diseases unrelated to heat stress, and literature reviews were excluded. Review articles were used only for contextualization, but were not part of the final analysis.

The selection process followed four stages: identification, screening, eligibility, and inclusion. In total, 487 studies were identified in the databases consulted. After removing 123 duplicates, 364 articles remained for title and abstract screening. At this stage, 287 works were excluded because they did not meet the criteria. There were 68 articles left for complete reading, of which 42 were excluded due to lack of data, inappropriate use of IRT, incompatible thematic focus, or unsatisfactory methodology. In the end, 23 studies met all the criteria and were included in the synthesis of this review (Table 1).

**Table 1:** Information about the articles used in the review.

Year	Author	Title	Thermal windows	Breed	Species	NA	Experimental design
2020	Diniz et al. (34)	Thermographic analysis applied to the body heat production of F1 H × Z cows managed in different microclimates	Right flank, left flank and ocular region.	Holstein × Zebu	*Bos taurus × Bos indicus*	48	A 2 × 2 factorial experiment evaluated microclimate (shaded vs. sun) and supplementation (with vs. without). Evaluation of environmental indexes.
2021	Stumpf et al. (41)	Different methods of assessing udder temperature through thermography and their relation with rectal temperature	Lateral portion of the udder.	Holstein	*Bos taurus*	38	A 6-day experiment with IRT to evaluate different methods of surface temperature measurement and its relationship with RT.
2021	Batista et al. (12)	Thermal images to predict the thermal comfort index for Girolando heifers in the Brazilian semiarid region	Regions of the head, trunk and limbs.	Girolando	*Bos taurus × Bos indicus* (crossbreed)	38	Experiment to correlate IRT with RT and THI and generate predictive models of thermal comfort.
2021	Campos et al. (42)	Thermography and physiology of stress in dairy calves in outdoor holding pens covered with geosynthetics	Head, neck, back, cannon and rump region.	Girolando	*Bos taurus* with crosses *Bos taurus × Bos indicus*	20	Experiment with geosynthetic covers in external stalls on the microclimate, evaluating thermal comfort and physiological responses using IRT to monitor thermal stress.
2022	Theusme et al. (35)	Prediction of rectal temperature in Holstein heifers using infrared thermography, respiration frequency, and climatic variables	Shoulder, belly, rump, leg, neck, head, forehead, nose, loin, leg, vulva, eye, flank.	Holstein	*Bos taurus*	200	Experiment to estimate RT through thermographic images, respiratory rate, and environmental conditions, adjusting and validating predictive models.
2022	Shu et al. (43)	Evaluation of the Best Region for Measuring Eye Temperature as the Predictor of Heat Stress in Dairy Cows	Eye region: medial corner, lateral corner, eyeball, entire eye, and lacrimal sac.	Holstein	*Bos taurus*	40	Experiment to identify the best region of the eye to measure ocular temperature by means of IRT, evaluating its relationship with heat stress.
2022	Bang et al. (44)	Application of infrared thermal technology to assess the level of heat stress and milk yield reduction of cows in tropical smallholder dairy farms	Region of the inner vulvar lip, surface of the base of the inner tail, eye area, muzzle, armpit area, area of the paralumbar fossa, anterior udder, posterior udder, anterior hoof, and posterior hoof.	Holstein	*Bos taurus*	344	This is a cross-sectional multicenter field study.
2023	Rodrigues et al. (45)	Thermal Signature: A Method to Extract Characteristics from Infrared Thermography Data Applied to the Development of Animal Heat Stress Classifier Models	Regions of the face, head, ribs and flanks of the animals.	Holstein	*Bos taurus*	18	Experiment using free-stall herd, monitored for 40 days, collection of RT and RR, meteorological variables and IRT in different body regions, to classify levels of heat stress, through artificial neural networks.
2023	Silva et al. (46)	Spatial modeling via geostatistics and infrared thermography of the skin temperature of dairy cows in a compost barn system in the Brazilian semiarid region	Head–back, cannon, udder	Holstein	*Bos taurus*	3	Thermal imaging (FLIR i60) at 9 a.m. and 3 p.m.; geostatistics and kriging to map Tpele and infer comfort;
2023	Brezov et al. (47)	Predicting the Rectal Temperature of Dairy Cows Using Infrared Thermography and Multimodal Machine Learning	Prediction of rectal temperature of dairy cows using infrared thermography.	Holstein	*Bos taurus*	295	IRT + RR + THI (and pulse in the subsample); models to predict RT.
2023	Mincu et al. (48)	Infrared thermography as a non-invasive method for evaluating stress in lactating dairy cows during isolation challenges	Infrared thermography as a non-invasive method to assess stress in lactating dairy cows during isolation challenges	Holstein	*Bos taurus*	20	Study with 20 Cows viewed by 240 min in stables, monitored via IRT
2020	Isola et al. (49)	Differences in body temperature between black-and-white and red-and-white Holstein cows reared on a hot climate using infrared thermography	Differences in body temperature between Dutch in hot weather by infrared thermography	Holstein	*Bos taurus*	30	seasonal comparison (cold vs. hot); Body IRT + RT
2022	Abduch et al. (50)	Effect of Thermal Stress on Thermoregulation, Hematological and Hormonal Characteristics of Caracu Beef Cattle	Body: left side of the body (from neck to rump), muzzle, loin and left eye.	Holstein	*Bos taurus*	91	field, before/after 8 h sun; IRT + RT + hematology + hormones
2023	Silva et al. (37)	Characterization of Thermal Patterns Using Infrared Thermography and Thermolytic Responses of Cattle Reared in Three Different Systems during the Transition Period in the Eastern Amazon, Brazil	anatomical regions of the head, axilla, flank, rump.	Nelore	*Bos indicus*	30	Variables evaluated: rectal temperature, respiratory rate, IRT (head, axilla, flank, and rump), and thermal comfort indexes.
2023	Silva et al. (36)	Thermographic Profiles in Livestock Systems under Full Sun and Shaded Pastures during an Extreme Climate Event in the Eastern Amazon, Brazil: El Niño of 2023	Environmental thermal mapping: Soil and tree area full sun areas, compost soil in sunny area, exposed soil and forage. Not applicable (environmental thermal assessment)	Nelore	*Bos indicus*	30	three systems (traditional, silvopastoral, integrated); thermal imaging camera to map area/pasture temperatures and infer animal comfort during *El Niño*
2023	Lei et al. (51)	Non-Invasive Biomarkers in Saliva and Eye Infrared Thermography to Assess the Stress Response of Calves during Transport	Eye region to assess the stress response of calves during transport	Nelore	*Bos taurus*	20	Pickups before transport, after transport and after rest
2023	Hoffman et al. (52)	Infrared thermography as an alternative technique for measuring body temperature in cattle.	Eye region, back of the ear and shoulder.	Nelore	*Bos taurus*	31	31 steers received LPS IV (0.25 μg/kg) to induce fever; continuous RT via rectal tube; ocular IRT (lacrimal caruncle) with FLIR E95 (ε = 0.89, ~1 m, angle 45–90 °); collections every 30–60 min until 12.5 h and then 18.5/24.5/36.5/47.5 h post-challenge.
2023	Romanello et al. (53)	Thermal comfort of Nelore (*Bos indicus*) and Canchim (*Bos taurus* × *Bos indicus*) bulls kept in an integrated crop-livestock-forestry system in a tropical climate	Microclimate evaluation: To evaluate the microclimate in a pasture system without shade and in a crop-livestock-forest integration system. Not applicable (environmental thermal assessment)	Nelore and Canchim	*Bos taurus e Bos indicus*	64	The experiment was conducted in a tropical region, São Carlos-SP, Brazil. The means were compared using Tukey's test (*p* < 0.05).
2025	Sousa et al. (54)	Non-Invasive Assessment of Heat Comfort in Dairy Calves Based on Thermal Signature	Head, rib and flank region.	Holstein	*Bos taurus*	10	Equipment: Clinical digital thermometer (RT). Stopwatch (respiratory rate). TESTO 875-2i thermal imaging camera (surface temperature). HOBO U12-012 recorder (air temperature, humidity and enthalpy).
2021	Idris et al. (10)	Relationship between ocular temperature by IRT of beef cattle and biological responses at high environmental temperatures.	Eye region	Angus	*Bos taurus*	24	Design: climatic chamber (exposure to heat; behavior, rumen/rectal and IRT of the eye)
2024	Blond et al. (55)	Influence of heat stress on body surface temperature and metabolic, endocrine and inflammatory parameters and their correlations in cows	Regions of the eye, ear, nose, forehead, whole head, abdomen, forelimbs, hind limbs, udder, and entire body.	Holstein	*Bos taurus*	90	Repeated measures on farm (May–July; Multi-region IRT + blood)
2024	Pereira et al. (56)	Predictive models for heat stress assessment in Holstein dairy heifers using infrared thermography and machine learning	Ocular region.	Holstein	*Bos taurus*	10	Climatic chamber (comfort vs. “heat wave”); construction/validation of classifiers using IRT
2025	Kim et al. (57)	AI-enhanced infrared thermography for reliable detection and spatial mapping of temperature patterns in calf eyes and muzzles	Eye and snout region of calves.	Holstein	*Bos taurus*	11	11 calves filmed three times in 4 week intervals, totaling 57 imaging sessions.

NA, Number of animals; F1, First generation; VS, Verses; H, Dutch; Z, Zebu; BGHI, Black Globe Humidity Index; RT, Rectal temperature; RR, Respiratory rate; IRT, Infrared thermography; THI, Temperature and humidity index; LPS, Lipopolysaccharide; h, Hours; IV, Intravenous; SP, São Paulo; P, *p*-value; μg, microgram; kg, kilogram.

These 23 articles also composed the comparative table, as they present homogeneous data that can be directly analyzed. However, to support the discussion and expand the theoretical framework, the review also used other relevant sources–including literature reviews and complementary articles that did not meet the inclusion criteria of the table, but contribute to the conceptual basis of the theme. Thus, considering the total set of works used in the construction of the review, 68 scientific papers were consulted.

For each selected study, data were extracted regarding the country of conduction, animal category, breed, type of thermal imaging camera, distance and angle of capture, experimental environment, thermal window evaluated, correlated variables and methodological limitations. These data were organized in a table in the section and served as a basis for the comparative analysis presented in the review.

The risk of bias and methodological quality of the included studies were assessed using predefined criteria inspired by the SYRCLE's Risk of Bias tool for animal studies, adapted to the characteristics of experimental cattle studies involving infrared thermography. The evaluation considered sample size adequacy, control of environmental variables, standardization of thermographic acquisition parameters, clarity of outcome measures, and completeness of reported data. Based on these criteria, studies were classified as presenting low, moderate, or high risk of bias. Most studies were categorized as having a moderate risk of bias, primarily due to limited sample sizes and variability in environmental and methodological control.

## 3 Results and discussion

A total of 23 studies met the eligibility criteria and were included in the final synthesis of this review. The selected studies were conducted under different climatic conditions, predominantly in tropical and subtropical regions, and involved dairy and beef cattle (*Bos taurus, Bos indicus*, and crossbreeds) managed in diverse production systems, including pasture-based, feedlot, and silvopastoral systems. The results are presented in a descriptive and comparative manner, focusing on the application of infrared thermography, the anatomical thermal windows evaluated, the integration with physiological and environmental indicators, and the main methodological characteristics and limitations reported across studies.

**Figure 1:**
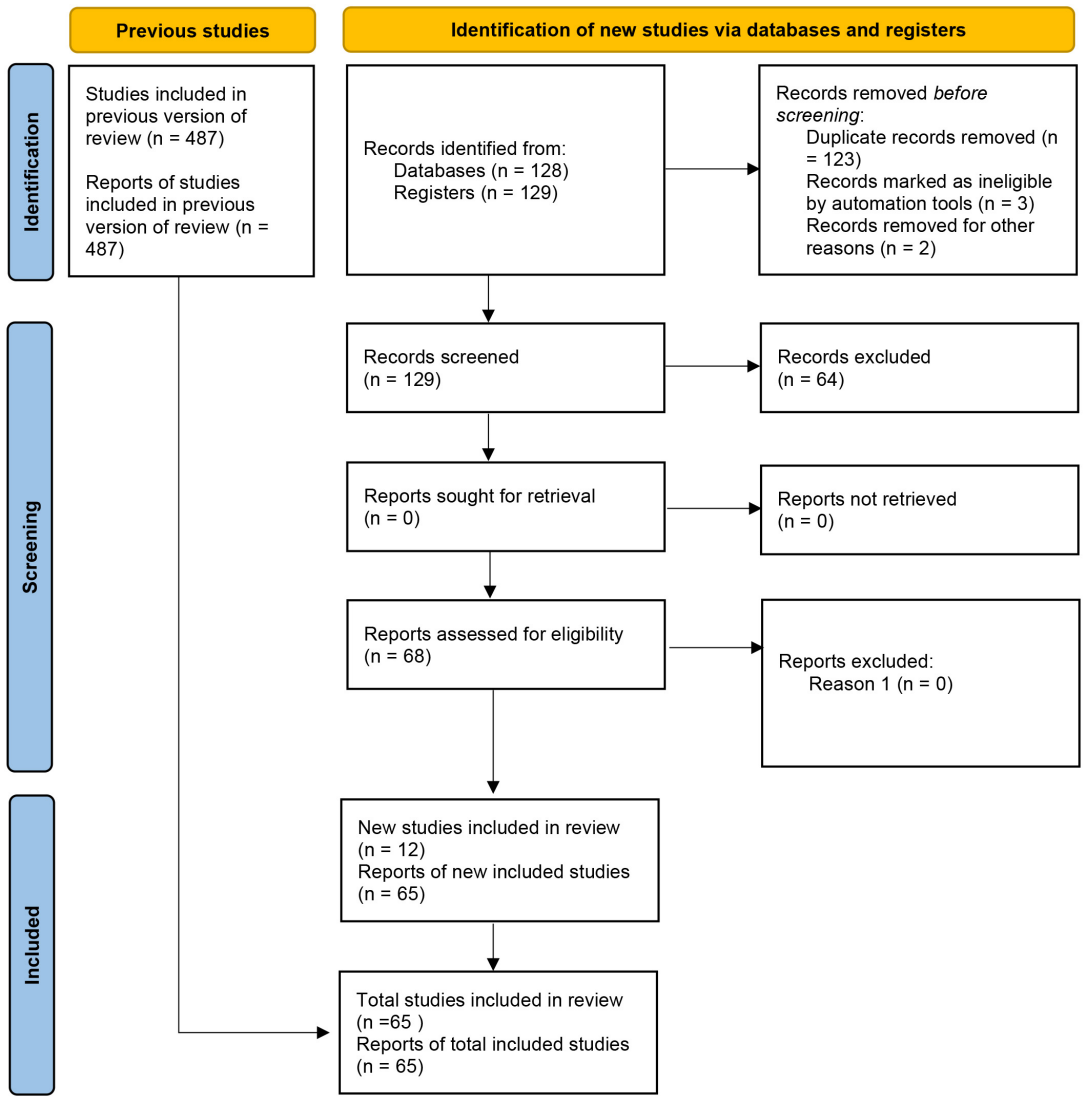
Flowchart used to select the articles used in the systematic review, following the PRISMA guidelines (16).

A flowchart (Figure 1) and PRISMA checklist will be included to detail all steps of the selection process, indicating the number of studies identified, excluded, and included in the final analysis (16).

### 3.1 Animal welfare: conceptual frameworks and scientific advances

Animal welfare evolved from the Brambell Report (17), which highlighted the importance of freedom of movement and natural behaviors, establishing the foundations of the modern concept (17, 18). This framework was expanded by the Farm Animal Welfare Council (FAWC), which consolidated the Five Freedoms, including the physiological, emotional, and behavioral needs of animals (19–21).

With the advancement of research, the principles of animal welfare were refined by the Welfare Quality^®^ project, which organized evaluation into four pillars: food, housing, health, and behavior, and established practical criteria applied internationally (19). This framework reinforced the multidimensional understanding of wellbeing, which involves reducing distress and promoting positive experiences (18, 19).

In practice, the evaluation depends on objective indicators. Animal-based measures, such as lameness, injuries, and body score, offer direct information about the physical and behavioral condition of cattle (22). At the same time, physiological parameters respiratory rate, body temperature, and cortisol help identify adaptation to heat stress (23).

The THI and BGHI are widely used to detect risk to wellbeing, especially in tropical regions, and become more accurate when combined with technologies such as infrared thermography. This integration makes it possible to monitor the microclimate and anticipate situations of heat stress (22, 23).

In this context, infrared thermography has stood out as a complementary and non-invasive tool for assessing thermal comfort and animal welfare. The integration of IRT with environmental indices enables microclimate monitoring and early identification of changes in thermoregulation, increasing the sensitivity of heat stress assessment and reducing the need for invasive management (18, 23).

### 3.2 Thermal comfort and thermoregulatory responses in cattle

Thermal comfort represents the dynamic balance between the animal and its environment, a condition in which homeostasis is maintained without additional energy expenditure (24). In cattle, this equilibrium occurs within the thermoneutral zone, typically described between approximately 5 °C and 25 °C for adult *Bos taurus* under moderate production levels. In contrast, *Bos indicus* breeds and their crossbreeds exhibit greater heat tolerance, maintaining physiological stability at environmental temperatures approaching 28–30 °C, depending on relative humidity and solar radiation intensity (24, 25).

When environmental conditions exceed these adaptive limits, particularly under high temperature, humidity, solar radiation, or inadequate ventilation, thermal balance is disrupted. Under such circumstances, heat stress develops and activates compensatory physiological responses aimed at restoring homeothermy, including increased respiratory rate, peripheral vasodilation, and alterations in metabolic activity (1).

As homeothermic animals, cattle rely on both behavioral and physiological strategies to maintain stable body temperature under challenging environmental conditions (24). Behavioral adjustments include seeking shade, increasing water intake, and reducing physical activity, while physiological responses involve increased respiratory rate, sweating, and peripheral vasodilation to facilitate heat dissipation (5, 25). These responses aim to preserve thermal balance and reduce the energetic cost of thermoregulation, thereby supporting productive performance and health (25).

Maintaining thermal balance reduces the energetic cost of thermoregulation, thereby supporting productive performance and metabolic efficiency (25). In tropical production systems, natural shading plays a crucial role in preserving this balance, as it mitigates solar radiation load and lowers surface and core body temperatures. Studies such as Reis et al. (3) demonstrate that shaded environments improve both physiological and behavioral responses, reinforcing the importance of microclimate management as a strategy to prevent heat stress.

Infrared thermography allows the non-invasive assessment of surface temperature patterns associated with peripheral blood flow and heat dissipation, complementing conventional indicators of thermal comfort (1, 26). When integrated with environmental indices, IRT enhances the monitoring of microclimatic conditions and supports the early identification of heat stress, especially in tropical production systems where factors such as solar radiation and shading strongly influence thermal load (3, 18, 23).

### 3.3 Heat stress

Heat stress occurs when cattle are unable to maintain homeostasis under conditions of elevated temperature, humidity, or excessive solar radiation, exceeding their heat dissipation capacity (4, 27). This imbalance between metabolic heat production and environmental heat loss compromises physiological stability, animal welfare, and productive efficiency, particularly in tropical systems (22, 27).

Initial responses are predominantly behavioral, including increased resting time, shade-seeking, reduced activity, and decreased rumination (7, 28). As thermal load intensifies, physiological mechanisms such as increased respiratory rate, sweating, and peripheral vasodilation are activated to enhance heat dissipation. Although adaptive, these responses are energetically costly and are commonly associated with reduced dry matter intake, impaired weight gain, and lower milk production (27, 28).

The magnitude of these effects varies according to production system and genetic background. High-yielding dairy cows are especially vulnerable due to elevated metabolic heat production, exhibiting reductions in feed intake and milk yield that may exceed 10% during heat waves, with persistent effects even after the return to thermoneutral conditions (25, 29, 30). Reproductive performance is likewise compromised, with decreased estrus expression and conception rates under elevated THI conditions (30, 31).

In beef cattle, heat stress reduces average daily gain and feed efficiency as dietary energy is redirected toward thermoregulatory processes (31, 32). Genetic background also plays a decisive role, as *Bos indicus* cattle demonstrate greater thermotolerance than *Bos taurus* due to morphological and physiological adaptations that enhance heat dissipation (32).

Given the dynamic and multifactorial nature of heat stress, infrared thermography has emerged as a strategic tool for monitoring thermal responses in cattle. By detecting changes in surface temperature associated with peripheral vasodilation, IRT enables early identification of heat stress before overt clinical signs appear (2, 7). When integrated into precision livestock systems, it supports adaptive management strategies such as shading, ventilation, and cooling interventions, contributing to improved welfare and production efficiency (8, 33).

### 3.4 Infrared thermography as a tool for the evaluation of thermal comfort

The IRT has gained prominence in veterinary medicine since its first records in the 1960s in horses, marking the beginning of its evolution to current precision livestock farming systems (2, 33). Its use in cattle has intensified in recent decades, with applications aimed at assessing thermal comfort, reproduction, early diagnosis of physiological disorders, and health monitoring (9). This advance is supported by the technique's potential to identify subtle surface temperature variations related to heat stress.

**Figure 2:**
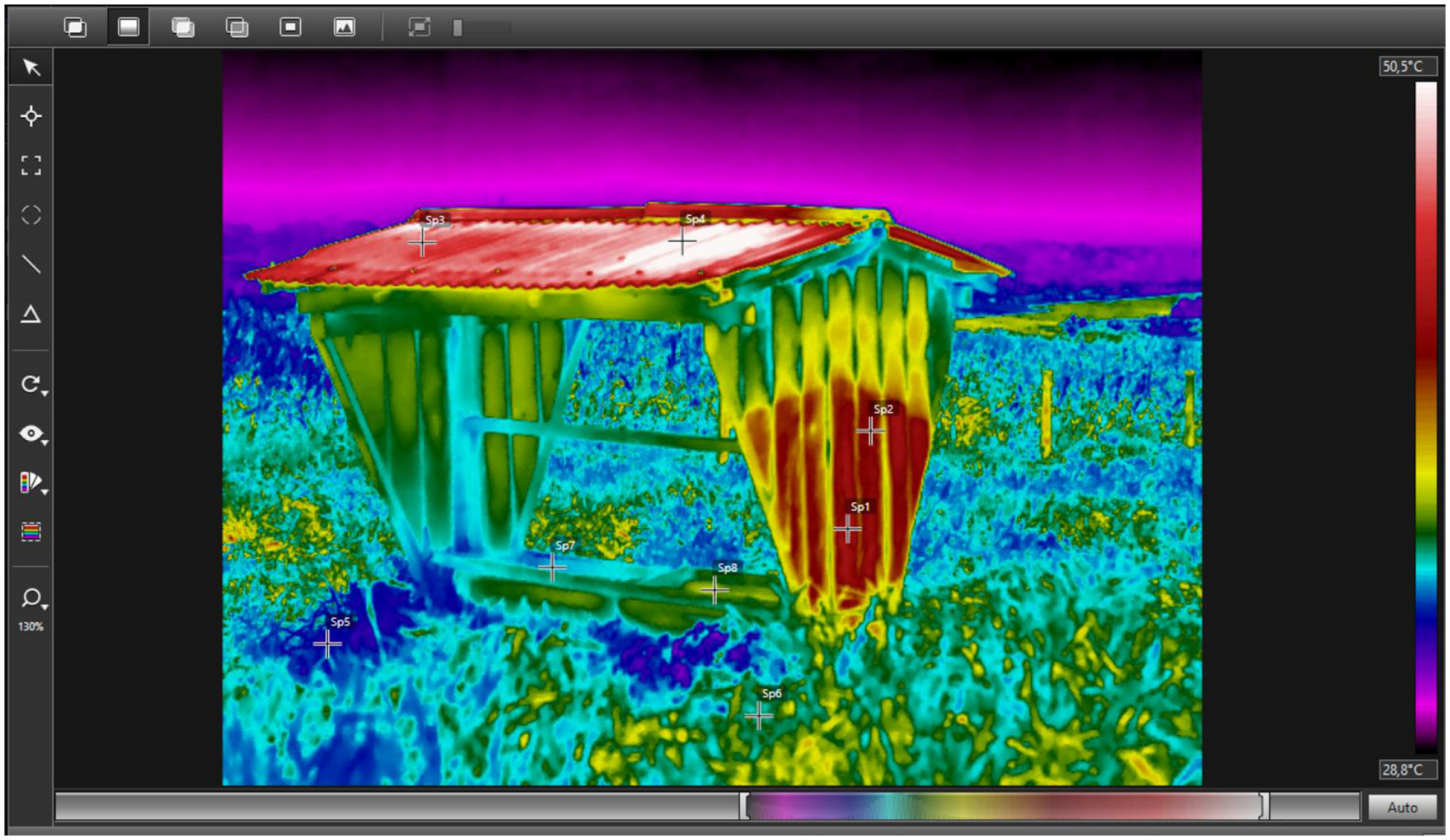
Infrared thermographic assessment of a feeding structure in a cattle production system. Surface temperatures recorded at different environmental points were: lateral wall (Sp1–Sp2), roof (Sp3–Sp4), forage (Sp5–Sp6), and feeder surface (Sp7–Sp8). Source: We monitored the targets by taking images with a near-infrared thermograph (FLIR T650sc), considering an emissivity of 0.95 because it is an open system composed of different materials. Thermograms were analyzed using the Flir Tools program, 6.3v, with the Rainbow HC palette chosen. Authors' own elaboration.

In addition to animal-related applications, IRT also allows the identification of critical heat accumulation points in structures built within livestock production systems, such as feeding facilities (Figure 2). Thermographic evaluation of the trough revealed higher surface temperatures on the roof (49.05 °C) and side wall (42.8 °C), while lower values were recorded on the trough surface (35.95 °C) and forage (34.4 °C). These thermal gradients indicate that upper structural elements act as important sources of environmental heat load.

The technique is based on the detection of infrared radiation emitted by the body surface and its conversion into thermal maps (2, 7). As a non-invasive method, IRT avoids the need for restraint and reduces the stress associated with handling, unlike measures such as rectal temperature and respiratory rate (33). Among the strengths of studies that employ this technique, the ability to monitor animals in different systems extensive, semi-intensive, and intensive systems, without compromising welfare, in addition to allowing repetition of measurements with minimal risk stands out.

Diniz et al. (34) reported significant differences in flank surface temperature under more challenging microclimatic conditions. In microclimate 2, right flank temperature was higher in supplemented animals (33.1 °C vs. 32.5 °C; *P* < 0.05), while rectal temperature remained unchanged across groups (mean 38.4 °C; *P* > 0.05). Correlations between rectal and ocular temperature were low to moderate (r ≈ 0.36–0.44; *P* < 0.01), indicating that surface measurements were sensitive to environmental variation without reflecting substantial changes in core temperature.

**Figure 3:**
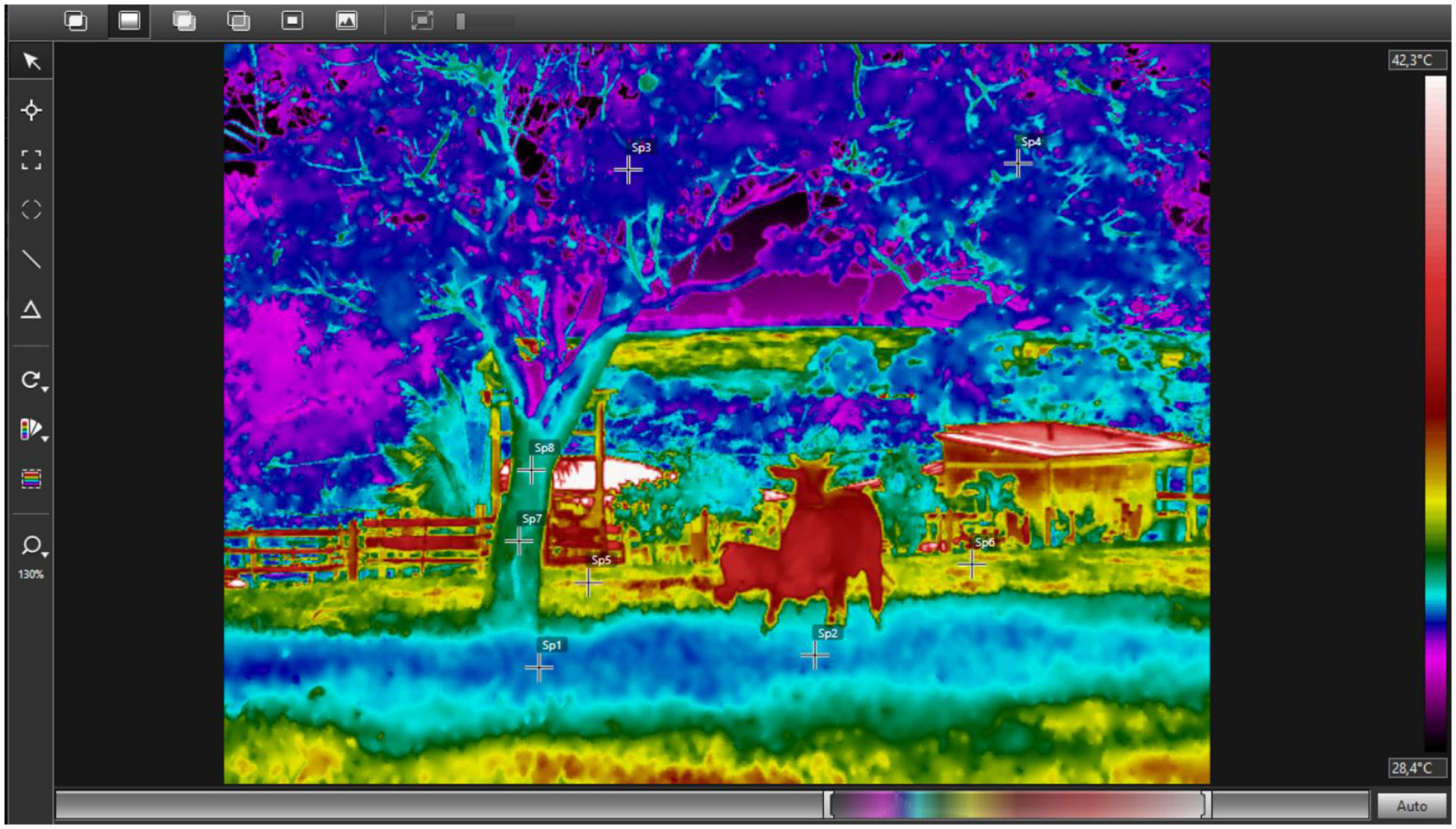
Infrared thermographic assessment of environmental microclimate in a pasture-based system with natural shading. Surface temperatures recorded at different environmental points were: shaded area (Sp1–Sp2; 31.5 °C), tree canopy (Sp3–Sp4; 31.15 °C), tree trunk (Sp7–Sp8; 32.2 °C), and forage exposed to direct solar radiation (Sp5–Sp6; 33.8 °C). Lower temperatures observed in shaded and canopy regions indicate reduced radiant heat load compared to forage under full sun exposure. Images were obtained using a near-infrared thermograph (FLIR T650sc) with emissivity set at 0.95, and thermograms were analyzed using FLIR Tools (v6.3) with the Rainbow HC palette. Source: Authors' own elaboration.

As an illustrative example, the thermographic assessment of the environment carried out in this study showed marked differences in surface temperature between the points analyzed (Figure 3). The shaded areas had an average temperature of 31.5 °C, as did the tree canopy with 31.15 °C, indicating greater efficiency in reducing the radiant heat load. The tree trunks had an intermediate temperature of 32.2 °C, while the forage exposed to direct solar radiation recorded the highest surface temperature of 33.8 °C. These results corroborate the findings in the literature by demonstrating that natural shading promotes more favorable microclimatic conditions, reducing the environmental heat load.

In addition, predictive approaches that integrate thermographic data with physiological variables and environmental indices, such as rectal temperature, respiratory rate, and THI, have achieved high accuracy in estimating thermal comfort, highlighting the potential of IRT based models for monitoring thermal status in different production systems (12, 35).

Nevertheless, when analyzing the literature cited, it is also observed that there are weaknesses and methodological limitations. Although Mota-Rojas et al. (2) and Ghezzi et al. (33) highlight the sensitivity of IRT to detect peripheral thermal changes, considerable variability exists in image acquisition protocols. Factors such as camera distance, emissivity settings, shading conditions, time of day, and angle of incidence are often inconsistently applied, limiting methodological standardization across studies. Barreto et al. (7), correlate IRT with environmental indices THI, BGHI, and Heat Load Index (HLI), but the capture parameters used differ from those employed by Zhang et al. (9) and Karvatte Junior et al. (8), which limits direct comparisons.

The IRT has also been applied in different production systems. In silvopastoral environments, Karvatte Junior et al. (8) demonstrated that the presence of shade significantly reduces surface temperature, with consistently higher canopy and pasture temperatures recorded under full sun conditions. Moderate to strong correlations between infrared surface temperature and environmental parameters (r = 0.45–0.78; R^2^ = 0.68–0.98) reinforce the measurable impact of shading on microclimatic thermal load. In regions of the Eastern Amazon, Cândido et al. (13) also reported distinct thermal profiles between full-sun and shaded pastures.

In young animals, the technique has been explored in the concept of thermal signature, identifying patterns that indicate early changes in comfort. Recent studies integrating IRT and artificial intelligence algorithms increase the accuracy of thermal classification (9, 28). Despite this, most of these approaches have technical limitations, such as dependence on camera resolution and image segmentation quality, aspects that are not detailed by the aforementioned authors.

In the face of climate change and the increase in heat waves (2, 28) IRT emerges as a strategic tool but it still lacks standardized protocols. Variables such as coat, skin color, dust, distance, and angle of capture remain critical factors that are not properly controlled or reported in part of the studies, which affects the reproducibility of measurements (10, 15). Thus, although the technique has great potential, its consolidation depends on methodological harmonization between studies, in addition to consistent integration with environmental indices and physiological measurements (36).

#### 3.4.1 Thermal windows in cattle: anatomical regions and applications in the evaluation of thermal comfort

Thermal windows represent anatomical regions with greater sensitivity to environmental variations, due to their high peripheral vascularization and direct role in heat dissipation. Mota-Rojas et al. (2) highlight the orbital and nasal areas as points that are highly responsive to heat stress, immediately reflecting changes in microcirculation. These authors also point out that these regions have good repeatability in the measurements, configuring a strong point in studies that use these areas as a reference. However, a recurring weakness is the lack of uniformity in the capture protocols, especially regarding the distance from the camera and the angle of measurement, which makes direct comparisons between the studies difficult.

Ghezzi et al. (33) reinforce the efficiency of the orbital regions and nasal cavity, identifying a strong correlation with environmental thermal load. In contrast, the authors applied the technique in specific microclimate conditions, which may limit the extrapolation of the results to rearing systems with greater environmental variability. Even so, the methodology used, with repeated measurements per animal, strengthens the robustness of the study, contrasting with studies that have a small sample size, making broader statistical analyses difficult.

Silva et al. (36), evaluating cattle in the Eastern Amazon, expanded the thermal windows analyzed, including flank, forehead, and hump. This expansion allows for more detailed thermal mapping, particularly in tropical production systems characterized by high spatial and temporal variability in solar radiation, humidity, and air temperature. However, the inclusion of multiple windows also introduces greater susceptibility to environmental interferences such as wind, solar radiation and dust representing a methodological challenge.

Batista et al. (12) identified regions such as the head, shin, back, and udder as highly sensitive to heat, allowing the development of predictive mathematical models. Even so, studies such as Silva et al. (36) and Ghezzi et al. (33) analyze different windows (orbital, nasal, and vulvar), evidencing the lack of consensus on which body region has better thermal stability under different contexts.

In young animals, the responsiveness of the thermal windows is even more evident. Silva et al. (37) observed that the ocular and nasal areas present rapid thermal oscillation in the face of changes in humidity and temperature, favoring their application in predictive models for calves. However, the study presents limitations typical of research involving neonates, such as small sample sizes and greater behavioral variability, which may introduce noise into the measurements and reduce observed thermal stability.

The appropriate choice of thermal windows is decisive for the accuracy of the analyses. The orbital and nasal regions stand out for their high correlation with heat stress and vasomotor sensitivity, being the most consistent in the studies by Mota-Rojas et al. (2), Ghezzi et al. (33) and Silva et al. (37). Areas such as the udder and vulvar are recognized for strong blood irrigation and rapid heat dissipation, although susceptible to interference from moisture or dust residues. Regions such as flank and forehead function as complementary points, supporting integrated analyses in tropical systems (37).

Although several thermal windows demonstrate potential for the evaluation of thermal comfort, the evidence presented by the authors indicates that the orbital and nasal regions are the most efficient, as they bring together greater thermal stability, rapid physiological response, and less behavioral influence. The use of additional windows can enrich the thermal profile, but requires more rigorous methodological care.

**Figure 4:**
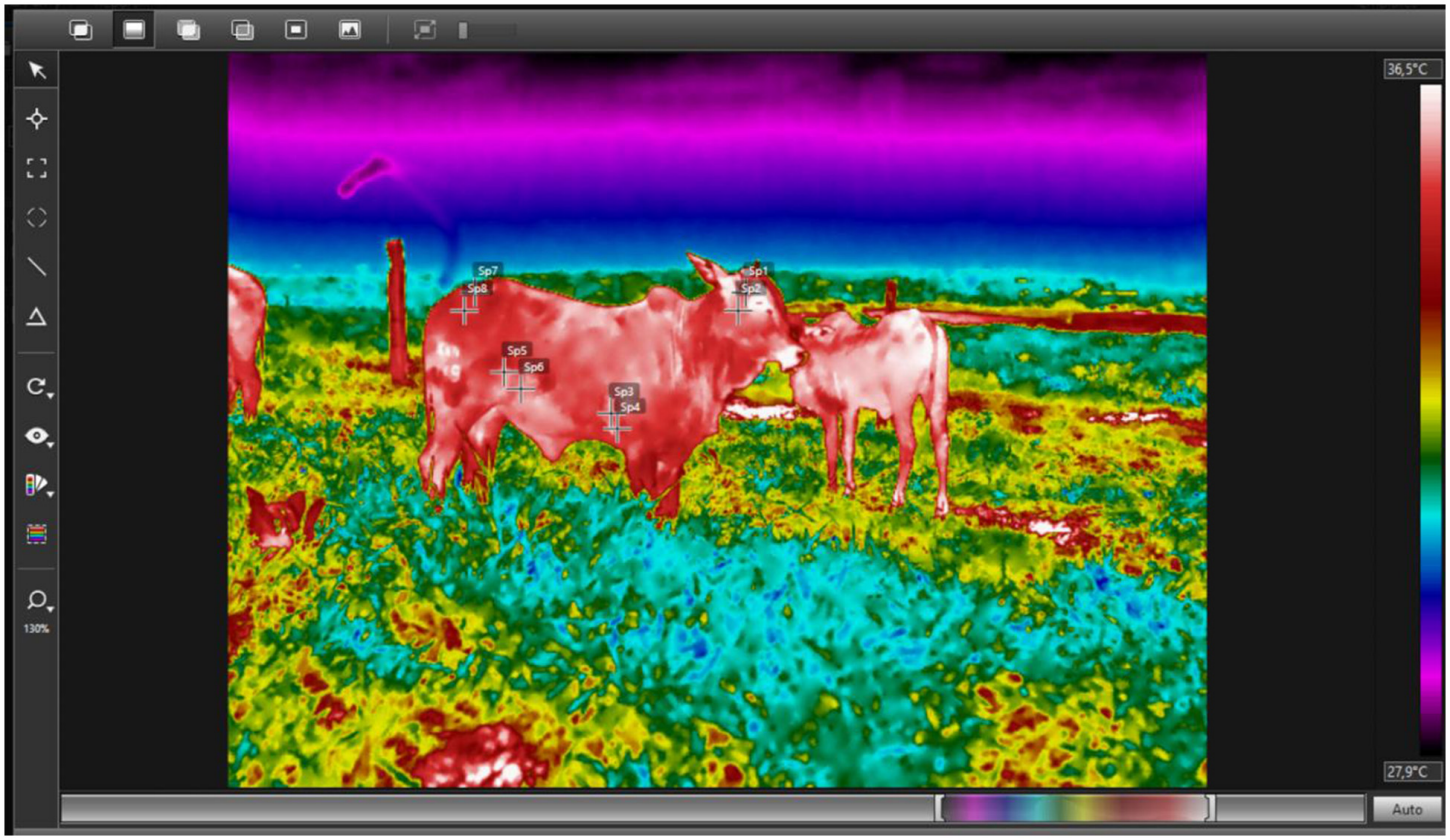
Infrared thermographic image illustrating different anatomical thermal windows commonly used in cattle for the evaluation of thermal comfort: head (Sp1-Sp2), axillary region (Sp3-Sp4), flank (Sp5-Sp6), and rump (Sp7-Sp8). We monitored the targets by taking images with a near-infrared thermograph (FLIR T650sc), considering an emissivity of 0.95 because it is an open system composed of different materials. Thermograms were analyzed using the Flir Tools program, version 6.3, with the Rainbow HC palette selected. Source: Authors' own elaboration.

Regions such as the ocular area, axillary region, flank, and rump exhibit distinct surface temperature patterns associated with peripheral blood flow and thermoregulatory responses. These anatomical sites are frequently applied in infrared thermography studies due to their sensitivity to heat stress and their relevance for assessing thermal comfort under different environmental conditions (Figure 4).

#### 3.4.2 Practical considerations for the application of infrared thermography in cattle

The application of IRT in cattle requires the adoption of rigorous methodological criteria, since the reliability, accuracy, and reproducibility of measurements depend directly on the standardization of collection procedures and the proper configuration of the equipment. The literature indicates that much of the variability observed between studies is associated with the absence of uniform protocols, especially with regard to environmental conditions, camera positioning, and the technical parameters used (2).

The time of acquisition of thermographic images is a determining factor for the correct interpretation of data. It is recommended that collections be carried out at standard times, preferably in the early morning and around noon, allowing the animal to be evaluated under different thermal conditions (37, 38). Measurements should be taken under natural or artificial shade, as direct sunlight can artificially raise the surface temperature and mask the animals' actual physiological state (4, 33).

Furthermore, environmental variables such as wind speed, relative humidity, and the presence of dust, mud, or moisture on the body surface should be considered and, whenever possible, recorded. Strong winds can accelerate heat dissipation by convection, while dirty or wet surfaces reduce skin emissivity, interfering with infrared radiation capture (2, 39).

The distance between the thermal imaging camera and the animal must be kept constant throughout the experiment, respecting the optical specifications of the equipment and the field of view necessary for the correct evaluation of the selected anatomical region (40). Although there is no single universally recommended value, most studies with cattle report the use of distances between approximately 1 and 3 meters, adjusted according to the camera resolution and experimental design (2, 5, 10–30, 30, 31, 31–58).

In more recent studies, Idris et al. (10) used shorter distances, ranging from 1.0 to 2.0 m, to maximize the spatial resolution of images in thermal stress assessments in cattle. Complementarily, Mota-Rojas et al. (2) emphasize that, regardless of the absolute value adopted, the distance must be standardized throughout the collection and adjusted to the technical characteristics of the camera, since variations in this parameter compromise the thermal scale and make it difficult to compare images.

The angle of capture also significantly influences the accuracy of measurements, so it is recommended that images be obtained at an angle of less than 45° to the body surface, minimizing reflections, geometric distortions, and loss of thermal information. In grazing systems, where animals move frequently, the use of fixed collection points, combined with repeated measurements, contributes to increasing the reliability of the data obtained (2, 40).

Proper calibration of the thermal imaging camera is essential for obtaining reliable images. The emissivity of bovine skin must be adjusted beforehand, commonly using a range between 0.95 and 0.98, varying according to the anatomical region evaluated and hair density. Incorrect adjustments of this parameter directly compromise the interpretation of surface temperatures (2, 5, 10–30, 30, 31, 31–59).

In this context, the consolidation of IRT as a robust tool for assessing thermal comfort in cattle depends on the development and adoption of uniform protocols. Technical training of operators and standardization of procedures are essential to reduce biases, increase the reproducibility of results, and expand the applicability of the technique in production systems, especially in tropical environments (37).

### 3.5 Infrared thermography as a complement to environmental indices

The IRT has been consolidated as a complementary tool to the traditional environmental indices used to assess thermal comfort in cattle. Studies suggest that the surface temperatures obtained by IRT are correlated with indicators such as the THI and the BGHI, which are widely used to estimate the environmental thermal load. Barreto et al. (7), observed a positive association between these indices and thermographic readings, emphasizing that IRT can contribute to more refined microclimatic characterizations, especially in tropical systems with high environmental variability (60).

A strength of this integrated approach is IRT's ability to capture thermal nuances that may go unnoticed by global environmental indices. While the THI and BGHI reflect average environmental conditions, the IRT evidences thermal differences in specific body regions that respond directly to radiant temperature, humidity, and wind. In contrast, most studies, including Barreto et al. (7), used relatively small animal samples and spot collections, which limits the generalizability of findings to larger herds and different climatic contexts.

Similar results were reported by Silva et al. (61) who studied Nellore cattle in wooded systems. In this study, lower respiratory rates, rectal temperatures, and surface temperatures were recorded compared to areas without shade, while the BGHI indicated conditions of mild to moderate heat stress. This contrast shows that, although environmental indices indicate an overall level of thermal load, IRT can reveal finer physiological responses associated with the local microclimate particularly in areas with abrupt variations in shading and ventilation. Despite this, the methodological differences between collections make it difficult to achieve a direct synthesis between studies, evidencing a methodological gap that has yet to be resolved.

In agroforestry systems, Ferreira et al. (62) applied IRT to tree canopies and observed positive correlations with environmental indices such as the THI, BGHI which integrate air temperature, humidity, and radiant heat load. These results reflect one of the strengths of IRT, i.e., its ability to integrate environmental and physiological variables into a single analytical framework. However, the study also revealed that interpretations can be affected by factors such as vegetation density, type of cover, and humidity conditions, which, when not controlled, introduce noise into the thermal data.

The potential of IRT also extends to the analysis of highly vascularized body areas. Mota-Rojas et al. (2) highlight that regions such as the ocular surface and udder have a thermal elevation consistent with the increase in THI, suggesting that these anatomical windows can function as physiological biosensors coupled to environmental conditions. This understanding is reinforced by Batista et al. (12), who state that the simultaneous use of IRT and environmental indices increases the accuracy of assessments in scenarios of high climate variability and can guide more effective management interventions.

Barreto et al. (7) demonstrated that small differences in shading, ventilation, and humidity can generate noticeable changes in the surface temperature of cattle, even when THI and BGHI values remain stable. These findings suggest that the IRT detects thermal nuances that environmental indices, as they are macro and standardized measurements, cannot capture with the same sensitivity. Ferreira et al. (62) also observed that thermography applied to vegetation in agroforestry systems reveal significant thermal differences that directly influence animal comfort, reinforcing the role of IRT as a microscale tool.

In scenarios where THI or BGHI indicate moderate thermal risk, IRT can help verify the presence of corresponding physiological responses and assess the effectiveness of mitigation strategies such as shading, irrigation, or ventilation (60). As described by Mota-Rojas et al. (2), vasomotor responses captured in thermal images serve as direct indicators of thermal adaptation, providing physiological insight beyond that offered by environmental indices alone.

In summary, IRT complements traditional environmental indices by offering a more detailed physiological and microclimatic perspective, increasing the sensitivity of thermal comfort diagnostics. However, the consolidation of this integration depends on the standardization of protocols, standardization of capture and analysis metrics, environmental control, and expansion of the samples studied elements that should be prioritized in future research to strengthen the practical application of IRT in tropical livestock.

### 3.6 Advantages of using infrared thermography in cattle

The IRT has important advantages for the evaluation of cattle, standing out for being a non-invasive, safe and containment-free technique. As it does not require physical contact, it reduces stress and the risk of accidents, ensuring greater wellbeing and allowing quick measurements of surface temperature (7, 37). This characteristic makes IRT especially useful in extensive systems, where direct management is limited and traditional methods are hampered by distance and herd behavior (63).

The technique also offers benefits for behavioral studies and for the identification of temperamental profiles. Cuthbertson et al. (63) showed that eye temperatures obtained by thermal imaging can serve as indicators of temperament, allowing predicting reactions to handling and associating stress patterns with the impact on meat quality. This integration between physiology and behavior makes IRT a valuable tool in precision livestock, extending its usefulness beyond thermal evaluation.

Another relevant advantage is the ability of IRT to detect changes related to heat stress at an early stage. As the images record instantaneous changes in body temperature, they allow quick responses in management, avoiding production losses and health risks (11). This sensitivity is strategic in tropical regions, where microclimatic oscillations can trigger thermal discomfort even when environmental indices appear to be moderate (33).

In this way, IRT also stands out for its ability to generate continuous information on animal welfare, helping to monitor herds without changing natural behavior. Idris et al. (10) and Silva et al. (37) reinforce that non-invasive technologies allow for more accurate assessments of thermal status, favoring management decisions, such as shading, ventilation, and stocking adjustments in real time. Cândido et al. (13) add that less human interference reduces biases in measurements, ensuring greater reliability to the results obtained.

Another important advantage of IRT is its applicability in different production systems, such as feedlots, pastures in full sun and wooded systems. In shaded areas, IRT can demonstrate significant reductions in surface temperature, evidencing the benefits of the microclimate provided by trees, and assisting in the validation of adaptive management strategies (64). Thus, the technique works as a comparative tool between systems, contributing to the choice of sustainable practices in tropical environments.

IRT enables analysis with a high level of detail analysis with a high level of detail, allowing the identification of specific thermal windows and the creation of predictive models of heat stress. When used in conjunction with environmental sensors and analysis algorithms, it becomes a robust tool for automated monitoring, with potential to support decision-making, reduce production losses, and improve the efficiency of cattle production systems (36).

### 3.7 Shortcomings in the use of infrared thermography in cattle

The IRT in cattle has limitations related to coat and skin color, which directly influence the accuracy of measurements. Dark-coated animals absorb more solar radiation and tend to have higher surface temperatures, while light coats reflect part of the heat, which can generate underestimated values (2). The thickness and density of the hairs also interfere with the thermal reading, making regions with less hair cover, such as the orbital and vulvar area, more reliable for monitoring (33).

Environmental conditions have a strong impact on the quality of thermal images. Direct solar radiation artificially raises body surface temperature, while shaded environments provide more stable and representative results (37, 56). Intense wind can accelerate convection heat dissipation, reducing captured surface temperatures. Factors such as dust, mud, or moisture also alter the reading, partially covering the infrared radiation emitted by the body (7).

Methodological aspects are another critical point for the accuracy of IRT. Readings obtained at angles above 45° or without standardization of the distance between the camera and the animal increase measurement variability, particularly in grazing systems where animal movement is frequent (36). These factors compromise the repeatability and comparability of thermographic data.

The calibration of the equipment is another determining factor. Incorrect adjustment of camera parameters, particularly skin emissivity values (0.95–0.98), has been reported as a recurring limitation that compromises the interpretation of surface temperatures in cattle studies (10). In addition, the camera needs to be configured according to the temperature and humidity of the environment to avoid artificial readings that do not correspond to the actual physiological state of the animals (15).

Finally, the literature points to the lack of methodological standardization as one of the most relevant limitations in the use of IRT. Studies vary in terms of the regions selected, the time of collection, the positioning of the camera, and the environmental parameters that must be recorded, making reliable comparisons and interpretations difficult (65). The consolidation of the technique depends on the creation of uniform protocols and the training of professionals to properly capture, interpret, and validate thermal images (36).

## 4 Final considerations

The use of infrared thermography has proven to be an efficient and non-invasive tool for assessing the thermal comfort of cattle. It enables the early identification of physiological changes associated with heat stress through different thermal windows, both environmental and anatomical. Among these, the ocular region and flank surface were consistently reported as responsive and practical indicators of thermal variation under different production conditions. Its ability to capture subtle variations in surface temperature increases the accuracy of analyses when used in conjunction with environmental indices and physiological indicators. However, limitations related to coat condition, climatic factors, and lack of methodological standardization remain challenges. Even so, the technique offers significant advantages for continuous monitoring of animal welfare, especially in tropical regions. Thus, IRT stands out as a promising resource for precision livestock farming, contributing to more sustainable and efficient systems adapted to current climate challenges.

## Data Availability

The original contributions presented in the study are included in the article/supplementary material, further inquiries can be directed to the corresponding author.
